# Psychological Education Health Assessment Problems Based on Improved Constructive Neural Network

**DOI:** 10.3389/fpsyg.2022.943146

**Published:** 2022-08-02

**Authors:** Yang Li, Jia ze Li, Qi Fan, Xin Li, Zhihong Wang

**Affiliations:** ^1^School of Administration, Nanjing Forest Police College, Nanjing, China; ^2^School of Foreign Studies, Nanjing University, Nanjing, China; ^3^Institute of Mental Health, Nanjing Xiaozhuang University, Nanjing, China

**Keywords:** LIWC dictionary, CNN, mental health assessment, assessment, psychological

## Abstract

In order to better assess the mental health status, combining online text data and considering the problems of lexicon sparsity and small lexicon size in feature statistics of word frequency of the traditional linguistic inquiry and word count (LIWC) dictionary, and combining the advantages of constructive neural network (CNN) convolutional neural network in contextual semantic extraction, a CNN-based mental health assessment method is proposed and evaluated with the measurement indicators in CLPsych2017. The results showed that the results obtained from the mental health assessment by CNN were superior in all indicators, in which F1 = 0.51 and ACC = 0.69. Meanwhile, ACC evaluated by FastText, CNN, and CNN + Word2Vec were 0.66, 0.67, 0.67, and F1 were 0.37, 0.47, and 0.49, respectively, which indicates the use of CNN in mental health assessment has feasibility.

## Introduction

Driven by the development of the mobile Internet, people have developed a variety of social networking platforms, the more influential ones include WeChat, Twitter, QQ, online forums, Facebook, Renren, Weibo, etc. They have become essential tools for people’s working lives. In terms of functional design, most social networking platforms support functions such as online communication, posting reviews, expressing emotions, and recording life. In particular, more and more users tend to express personal emotional thoughts or communicate their psychological state on social networking platforms, through which they can seek help when necessary. Therefore, social networking platforms have also become an important tool for studying mental health problems. By studying the information on social networking platforms, we can identify whether users have mental health problems such as self-harm and depression, and we can further trace the underlying causes of mental health problems. As deep learning algorithms have matured, the field of natural language processing has begun to apply deep learning algorithms more widely and has made positive progress. For example, Nanni Loris et al. used convolutional neural networks to classify sentiments of sentences and determine the positive and negative polarity of sentences by extracting n-gram features through multiple channels. The advantage of constructive neural network (CNN) is that it can capture the features of local sequence information, but it also has the obvious disadvantage that its convolutional kernel size is fixed, and thus it cannot model longer sequence information (Liu et al., 2021; Nanni et al., 2021; Wang et al., 2021). In this regard, Zhou used the Bi-LSTM structure to replace the convolution and pooling process of Text CNN to express the contextual information of text and extracted the valuable information in text by two-dimensional convolution and pooling in two dimensions, and this method effectively improved the accuracy of text classification for various application scenarios such as news classification, question classification, and sentiment classification (Zhou et al., 2016; Shaikh et al., 2021). Liu used a mathematical model based on deep learning algorithms for the personality prediction of social network users (Joulin et al., 2016; Cui and Zang, 2021; Kanekar et al., 2021; Ren et al., 2021). Yates analyzed the process of detecting mental health problems of online forum users and concluded that feature construction is the most tedious task in the detection process, for which convolutional neural networks were invoked for feature construction, which greatly simplified the workflow (Yates et al., 1709; He et al., 2021). This study believes that the automatic assessment of mental health can be strengthened by combining the analysis of relevant data on social platforms. The automatic assessment of mental health mainly includes the detection and analysis of depression and suicide intention. In addition, the analysis of personality closely related to mental health problems and the analysis of reasons for emotional changes are helpful to the automatic assessment of mental health. In view of the need for psychological education health assessment, a set of mental health assessment models based on the linguistic inquiry and word count (LIWC) dictionary and CNN is constructed, and the feasibility of this model is demonstrated.

## Basic Methods

### Basic Principles of Constructive Neural Network

#### The Structure of Constructive Neural Network

**FIGURE 1:**
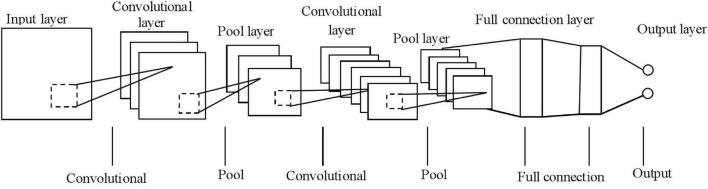
Architecture of constructive neural network (CNN).

In the CNN structure, the first layer is the input stage, the second and third layers are the first feature extraction stage, the fourth and fifth layers are the second feature extraction stage, and the sixth and seventh layers are the classification stage, in which the classifier is a two-layer fully connected neural network. The CNN architecture is shown in Figure 1 below.

In terms of construction, CNN contains seven layers, namely, the input layer, convolutional layer, downsampling layer, fully connected layer, and output layer. Generally, there are one to three feature extraction stages in the CNN implementation process, after which the extracted feature data is delivered to the classifier. The 7-layer network structure shown in Figure 1 above contains 2 feature extraction stages corresponding to a 4-layer network, while the classification stage occupies a 2-layer network, and this architecture design can meet the classification requirements in most application scenarios.

The first layer is the input layer, which only accepts images of standard size N*N.

The second and fourth layers are convolutional layers, which perform convolutional operations on the previous layer to obtain multiple feature maps. The upper layer performs convolutional operations with multiple trainable convolutional kernels of size k*k, plus a bias, and acquires a feature map of size (*N - k* + 1)*(*N - k* + 1) and the same number as the convolutional kernels after an excitation function. The equation for the above process is as follows (Caldelli et al., 2021; Khaydarova et al., 2021; Kumar and Hati, 2021; Moccia et al., 2021; Shi et al., 2021; Szajna et al., 2021):


(1) $$x_{j}^{l}=f\,\left(\sum_{\text{i}\in M_{j}}x_{i}^{l-1}\times k_{i\,j}^{l}+b_{j}^{l}\right)$$

In which, *k* denotes the convolution kernel, *l* denotes the number of layers, *b* denotes the bias, and *M*_*j*_ denotes the *j*th feature map.

The third and fifth layers are down-sampling layers, which sub-sample each feature map of the previous convolutional layer. This process reduces the resolution of the feature maps, effectively shortening the data processing time while ensuring that valuable information is not lost. The maximum or weighted summation operation is performed on the neighborhoods of size m*m within each feature map, and a bias is added to obtain the same number of feature maps of size (*N* - *k* + l)/m after an excitation function as at the time of input. The equation for the above process is as follows:


(2) $$x_{j}^{l}=f\,\left[\beta_{j}^{l}\,\text{down}\,\left(x_{j}^{l-1}\right)+b_{j}^{l}\right]$$

In which, down0represents the downsampling function, and β, *b* represent the feature maps of each output, respectively.

The sixth layer is the classifier, which connects the feature maps extracted from the previous layer to build feature vectors for learning.

The seventh layer is the output layer, which outputs the corresponding tags.

#### Constructive Neural Network Training Process

##### Input

Build training sample set *X* = {*x*_i_}, label *T* = {*t*}_i= 1_.

##### Initialization

Set the number of hidden layers, the learning rate, the number of neurons in the convolutional and downsampling layers, initialize the connection weights and biases between the networks, and choose a smaller random number.

Note: The training samples are denoted as <*x*, *t*>, where *x* denotes the network input value vector, and *t* denotes the target output value.

##### The Training Process

Propagate the input forward along with the network. One sample is pulled from within the dataset and imported into the CNN, *l* denotes the current layer, and the output of the current layer is formulated as:


(3) $$x^{l}=f\,\left(u^{l}\right),\text{with}\,u^{l}=w^{l}\,x^{l-1}+b^{l}$$

In which, *f* denotes inputting activation function, *b* denotes the bias, and *w* denotes the weight matrix.

Each layer processes and passes the data until the final layer outputs the data as follows:


(4) $$\text{output}=f_{n}\,\left(\cdots \,\left(f_{2}\,\left(f_{1}\,\left(x_{i}\,w_{1}\right)\,w_{2}\right)\right)\,\cdots \,w_{n}\right)$$

Propagate the error backward along with the network.

Sum the output units in the network and evaluate the error by referencing the squared error cost function, and the equation is as follows:


(5) $$E_{d}=\frac{1}{2}\,\sum_{k}^{n}{\left(t_{k}-o_{k}\right)}^{2}$$

In which, *n* denotes the set of each output unit, *E*_*d*_ denotes the error of the training sample *d*, *o*_*k*_ denotes the output value of unit *k* in the training sample *d*, and *t*_*k*_ denotes the target value of unit *k* for the training sample *d*.

For each output cell *k*, the error term δ_*k*_ for the current layer *l* is:


(6) $$\delta_{k}\leftarrow f^{\prime }\,\left(u^{l}\right)\,\left(t_{k}\,o_{k}\right)$$

Solve the error term δ_*k*_ for each hidden layer cell in the network as follows:


(7) $$\delta_{h}\leftarrow f^{\prime }\,\left(u^{l}\right)\,\sum w_{h\,k}\,\delta_{k}$$

The arithmetic of δ_*k*_ in the above equation is $\delta^{1}=w^{l+1}\delta^{l+1}•f^{1}\left(u^{1}\right)$.

Update each network weight *w*_μ_ as follows:


(8) $$w_{\mu }\leftarrow w_{\mu }+\Delta \,w_{\mu }$$

(9) $$\Delta \,w_{\mu }=-\eta \,\frac{\partial E_{d}}{\partial w_{j\,i}}=\eta \,\delta_{j}\,x_{j\,i}$$

### Linguistic Inquiry and Word Count Dictionary

LIWC is one of nature language processing (NLP) technologies that can analyze various texts through computer programs. It can make a quantitative analysis of the text content and calculate the different categories of words in the imported text file, especially for psychological words, which can calculate the percentage of use of such words in the entire text. After more than a decade of development, revision, and expansion, LIWC has become increasingly stable, and it mainly includes two parts, namely, the program body and the dictionary. Among them, the core is the dictionary, and the words in the text can be compared with the dictionary one by one, and then the word frequency results of various words are output. The current LIWC has a total of 80-word categories and about 4,500 words.

For existing LIWC-based text analysis methods, although this method is simple and effective, it does not consider the order between words and the contextual information of words, which will lead to large errors in text analysis. For example, when counting emotional words, the LIWC dictionary can only judge the category of emotional words but cannot accurately judge the specific emotional polarity of them. In addition, negative words and degree words in the context of emotion words cannot be accurately analyzed.

## Automatic Assessment Model of Mental Health Based on Constructive Neural Network

In order to make full use of psychological feature information in LIWC, a set of convolutional neural networks based on LIWC (LIWC-CNN) is constructed (Tanana et al., 2021). First of all, the LIWC dictionary is used to determine the distinct degree of a certain word class on the mental health of posting users. Then the convolutional neural network is guided to extract the key psychological features in posts, which is used to train the automatic assessment model of mental health. In addition, LIWC can accurately represent the mental health status, and CNN has explained the context information of the words in the post (Tay, 2021), so the combination of the two can accurately assess the mental health status reflected in the post.

### Weight Calculation of Word Classes

In 80 word classes in the LIWC dictionary, there are 16-word classes belonging to the descriptive word classes without actual semantics, such as the number of words per sentence, the total number of words, and so on, which are not the focus of this manuscript. The paper only studies the 64 semantically valid word classes in LIWC, including social process words, cognitive process words, negative words, personal pronouns words, and others. Considering that words in different parts of speech play different roles in identifying users’ mental health status, this paper determines the weight of each word class through standard deviation analysis, and the implementation process is as follows (Pandi et al., 2018):

Step 1: determine the word frequency of each word class. The frequency of different word classes in the posts of green, red, crisis, amber, and others is analyzed by using the language feature screening procedure.

The following linguistic features are screened by means of SD analysis:

Solve for the frequency of the word class. The frequency of occurrence of each word class in the LIWC dictionary in a sample of different mental health categories was solved respectively and expressed in mathematical language as follows: for sample $\left\{p_{i}=<t_{1}^{i},t_{2}^{i},\ldots ,t_{k}^{i},\ldots ,t_{n_{i}}^{i},>,\text{i}=1,\ldots ,|D|\right\}$ in the training set, |*D*| denotes the number of the samples, *n*_*i*_denotes the text length of the sample*p*_*i*_, $t_{k}^{i}$ denotes the *k*th word in the sample *p*_*i*_, the frequency of occurrence*TF*(*l*,*c*) of word class *l* in sample class *c* ∈ {*crisisredambergeren*} is as follows:


(10) $$\begin{array}{c} T\,F\,l,c=\frac{\sum_{i=1}^{\left|D\right|}\sum_{j=1}^{n_{i}}1_{l}\,\left(t_{j}^{i}\right)\cdot 1_{c}\,\left(p^{i}\right)}{\sum_{i=1}^{M}n_{i}\cdot 1_{c}\,\left(p^{i}\right)}\\ A\,\left(x\right):=\left\{ \begin{array}{c} 1\\ 0 \end{array}\right.\,\,\begin{array}{c} i\,f\\ i\,f \end{array}\,\,\begin{array}{c} x\\ x \end{array}\,\begin{array}{cc} \in A & \\ \notin A & \end{array} \end{array}$$

Solve for the SD of the word class. The results of solving for the standard deviation of the frequency of each word class in the sample across mental health categories highlight the differences between the word classes, and larger standard deviations imply that the word class is effective in distinguishing the mental health category to which the sample belongs. Considering the significant differences in the frequency of occurrence of different word classes in the dataset, the peak frequency of occurrence of the word class in each mental health category was used for normalization when solving for the standard deviation. The equation is as follows:


(11) $$\delta_{t}^{2}=\sum_{c}{\left[T\,F\,\left(l,c\right)-\mu \right]}^{2}$$

(12) $$T\,F^{\prime }\,\left(l,c\right)=\frac{T\,F\,\left(l,c\right)}{\max\left[T\,F\,\left(l,c\right)\right]}$$

(13) $$\mu =\frac{\sum_{c}T\,F^{\prime }\,\left(l,c\right)}{\sum_{c}l}$$

In which, μ denotes the average of the normalized frequency of word class *l* over the four types of samples, and *TF*′(*l*,*c*) denotes the normalization of *TF*′(*l*,*c*). $\delta_{t}^{2}$ represents the standard deviation of the word *l* belonging to the speech class *t*.

Word class screening. The top-k LIWC word classes with the highestδ_*1*_ are selected as linguistic features. According to the experimental results, the best classification results can be harvested when *k* = 28 is set, so the 28 most discriminative word classes from the 64 LIWC word classes are selected as linguistic features. The feature values corresponding to word class *l* in the linguistic features of the training sample or test sample *p*_*i*_ are *LGF*(*l*,*p*_*i*_) as follows:


(14) $$L\,G\,F\,\left(l,p_{\text{i}}\right)=\frac{1}{n_{i}}\cdot \sum_{j=1}^{n\,i}1_{l}\,\left(t_{j}^{i}\right)$$

Step 2: Solve the SD of the word frequency of each word class. The word frequency of different parts of speech in four posts is obtained in step one, then the SD of each word class word frequency is calculated. At the same time, the maximum value is used to perform normalization processing to evaluate the differences in LIWC word class in different mental health categories. That is to say, with the increase of the standard deviation, the differentiation degree of this word class for the mental health category is greater. The top 10 LIWC word classes with SD δ_*l*_ are listed in Table 1.

**TABLE 1:** Top 10 speech classes with standard deviation of word frequency.

Ranking	Word class	Standard deviation	Ranking	Word class	Standard deviation
1	Relig_c	0.4269	6	Money_c	0.3304
2	Friends_c	0.3965	7	Assent_c	0.3237
3	Death_c	0.3867	8	Anger_c	0.2796
4	You_c	0.3658	9	Ingest _c	0.2823
5	We_c	0.3469	10	Sexual _c	0.2502

Step 3: Adjust the weight of words in the post. According to the attribution of words in the post in the LIWC category, the weight of words is adjusted to give a higher weight to those words that can clearly distinguish the post category. If the word in the post does not exist in LIWC, the weight of the word can be set to 1. The formula for the weight *a*_*i*_ of the word *i* is as follows:


(15) $$a_{i}=\left\{ \begin{array}{cc} \frac{\exp\left(\delta_{l,i}\right)}{\sum_{l}\exp\left(\delta_{l,i}\right)}+1 & i\,f\,i\in l\\ 1, & i\,f\,i\notin l \end{array}\right.$$

where, δ_*l,j*_ refers to the SD of the word *i* belonging to the word class *l*. If the word *i* belongs to more than one word class, the value with the maximum SD of the word class should be selected.

### The Construction of the Mental Health Assessment Model Based on Constructive Neural Network

**FIGURE 2:**
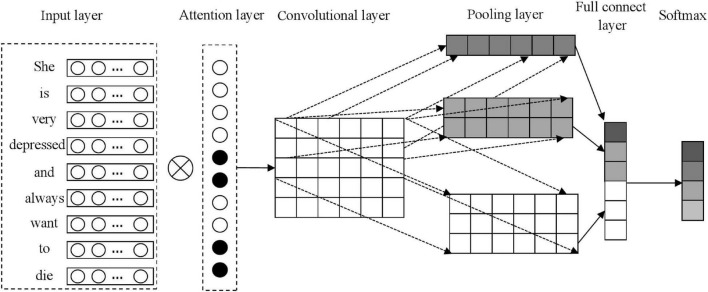
The linguistic inquiry and word count (LIWC)-CNN model architecture.

As a typical deep learning algorithm, CNN integrates convolution kernel operations, which can effectively increase the model depth. The traditional CNN consists of a pooling layer, a convolutional layer, and other parts. Here, the convolutional layer uses special convolution kernel operations to determine the local feature map of the text, and the pooling layer is used to locate effective features. To realize the automatic assessment of users’ mental health, the advantages of Kim architecture and the LIWC dictionary are comprehensively applied to construct the LIWC–CNN model. The LIWC–CNN model architecture is shown in Figure 2.

Combined with the figure above, the LIWC-CNN model contains four parts, specifically:

Input layer: Decompose the post into multiple sequences with *n* words. If the number of words is less than *n*, it needs to use special markers to complete the post. If the number of words is greater than *N*, it needs to intercept the front *n* words as a substitute. Then, the Word2Vec model is adopted to train the word vector model, so as to represent the post in the form of a word vector matrix *X*, namely, $X=\left[{\vec{x}}_{1},{\vec{x}}_{2},\ldots ,{\vec{x}}_{i},\ldots ,{\vec{x}}_{n}\right],{\vec{x}}_{i}\in R^{1\times d}$. ${\vec{x}}_{i}$ refers to the vector of the *i*th word, and *d* refers to the dimension of the word vector.

Attention layer: In combination with the analysis results, the attention layer further improves the recognition performance of posts by utilizing the ability of the LIWC word class to distinguish samples. After determining the weight of words in the post, it is weighted with the word vector matrix *X* of the input layer to construct a new word vector matrix *M*. The formula is as follows:


(16) $$\begin{array}{c} M=\vec{\alpha }\cdot \vec{X}\\ \vec{a}=<a_{1},\ldots ,a_{i},\ldots ,a_{n}>\\ \vec{X}=<{\vec{x}}_{1},{\vec{x}}_{2},\ldots ,{\vec{x}}_{n}> \end{array}$$

Convolutional layer: Utilize the convolutional kernel of each window to obtain the local semantics of the text sequence, so as to realize feature detection. In operation, the convolution kernel of sliding window *h* is selected to obtain the local semantics of sentences in posts, and the word vector matrix is constructed by splicing semantic information. Decompose the sentence with *n* words into < *x*_0:*h*−1_,*x*_1:*h*−1_,…,*x*_*i*:1 + *h*−1_,…,*x*_*n*−*h* + 1:*h*_ >, and apply the following Equation 16 to perform a convolution operation on each component *x*. At the same time, call the activation function δ(*x*) to realize the de-linearization process, so as to solve the feature vector ${\vec{g}}_{j}$. Finally, the feature matrix *G* that can present global semantics can be obtained by splicing the feature vector, as follows:


(17) $${\vec{\text{g}}}_{f}=\partial \left[f\,\left(w\cdot X_{j:j+h-1}+b\right)\right]$$

(18) $$\delta \,\left(x\right)=\frac{1}{1+e^{-x}}$$

(19) $$G={\vec{\text{g}}}_{1}⊕{\vec{\text{g}}}_{2}⊕\ldots \,{\vec{\text{g}}}_{j}⊕\ldots ⊕{\vec{g}}_{n-h+1}$$

Among them, *f*(⋅) refers to the convolution operation function, *w* ∈ *R^h×k^* refers to the weight parameter of the convolution kernel, *g*_*i*_refers to the eigenvalue obtained by the convolution operation against the component *X*_*i:i+h–1*_, *b* ∈ *R* refers to the bias value, and ⊕ refers to the vector splicing action.

Pooling layer: Use the appropriate pooling functions to find out effective feature values in global semantic information, so as to achieve the target task more efficiently. Max function is most commonly used, as follows:


(20) $$\vec{\overset{⌢}{G}}=\left[\max\left({\vec{g}}_{1}\right),\max\left({\vec{g}}_{2}\right),\ldots ,\max\left({\vec{g}}_{i}\right),\ldots ,\max\left({\vec{g}}_{n-h+1}\right)\right]$$

Full connect layer: Feature vector is obtained through the above steps, which is set as semantic feature information $\bar{F}$ representing the original text, and then softmax function is called to solve the post-classification result *y*, as follows:


(21) $$\vec{F}=\sum_{i=1}^{n-h+1}\left(w\cdot {\vec{\overset{⌢}{G}}}_{i}+{\vec{b}}_{i}\right)$$

(22) $$y=\text{soft}\,\max\left(V\cdot \vec{F}+b\right)$$

(23) $$\text{soft}\,\max\left(x\right)=\frac{e^{x}}{\sum_{k=1}^{K}e^{x}}$$

## Model Evaluation and Experimental Analysis

### Experimental Data and Evaluation Indicators

The data set adopts social posts provided by the CLPsych2017 Workshop and some posts in the emotion column of Sina Weibo. After selecting the sample data, the data content is preprocessed, such as deleting the information content that cannot be decoded and recognized, data conversion, and evaluation indicators such as Urgent F1, Non-Green F1, All F1, Flagged F1, and others are used to evaluate the model performance.

Among them, for different types of posts, after analysis, it can be known: For green post, it does not show any signs of self-harm tendencies; for amber posts, it contains clues that are likely to show signs of mental health problems; for red post, it indicates that the user is suffering from a more serious problem and needs the help of the forum moderator as quickly as possible; for crisis post, it shows a strong tendency to self-harm. The statistics found that the negative emojis in the crisis and red samples are mostly negative, and the emojis in green sample are mostly positive. Therefore, the emojis that only appear in the crisis or red samples (collectively referred to as the urgent class) are uniformly marked as negative, the emojis that appear only in the green or amber samples (collectively referred to as non-urgent classes) are uniformly marked as positive, and if the emojis appear in both the urgent class and non-urgent class, they are uniformly marked as neutral.

The posts are initialized with the Word2Vec word vector, and the cross-entropy loss function is minimized by the back propagation mode. What’s more, a set of optimal model parameters is obtained. To avoid the overfitting phenomenon, L2 regularization is performed forhe weight coefficient W (Ahmad et al., 2017; McHaney et al., 2018; Razavi et al., 2021; Wiggins et al., 2021).

### Data Preprocessing

The basic operations of data preprocessing are data conversion, emoji labeling, and data filtering. Where data conversion refers to link replacement and case conversion. The former represents the unified replacement of all links in the post with “URL,” so as to cut off the connection with the link object. Data filtering represents the removal of non-digital, non-English, and non-punctuation symbols that are difficult to be decoded and recognized in the sample. Emoticon labeling refers to the emoticons in samples being found out and using them to analyze users’ emotional states. According to statistics, there are a large number of emoticons reflecting negative expressions in the red and crisis samples, while the green samples are filled with emoticons reflecting positive expressions. Therefore, the emoticons in the urgent samples are uniformly labeled as negative and those in the non-urgent samples as positive. If a certain type of emoticon exists in both samples, they are uniformly labeled as neutral.

It is important to note that the stop word removal operation is usually not performed during preprocessing. The reasons are as follows: first of all, the distribution of stop words is uneven in each sample, which can reflect the users’ usage habits and indirectly help the classification process. Then, the stop words may change the emotional orientation displayed by the text content. For example, the apparent proportion of “not” in the crisis sample is significantly higher than that in the green sample.


(24) $$Loss=\frac{1}{N}\sum_{i=1}^{N}\left[y_{i}\ln{\overset{⌢}{y}}_{i}+\left(1+y_{i}\right)\ln\left(1-{\overset{⌢}{y}}_{i}\right)\right]+\frac{1}{2}\lambda ||\omega |{|}_{2}^{2}$$

### Parameter Settings

After obtaining the sample data, the relevant verification experiments are carried out immediately. The hyper-parameter values of the CNN model are shown in Table 2.

**TABLE 2:** Model hyper-parameter setting.

Hyper-parameter	Description	Value
D	Word vector dimension	128
N	Learning rate	0.001
H	Convolution kernel sliding window size	23,5,7
Batch size	Batch size	50
P	Dropout rate	0.1
M	Number of convolution kernels	48
∧	Regularization coefficient	0.1

**FIGURE 3:**
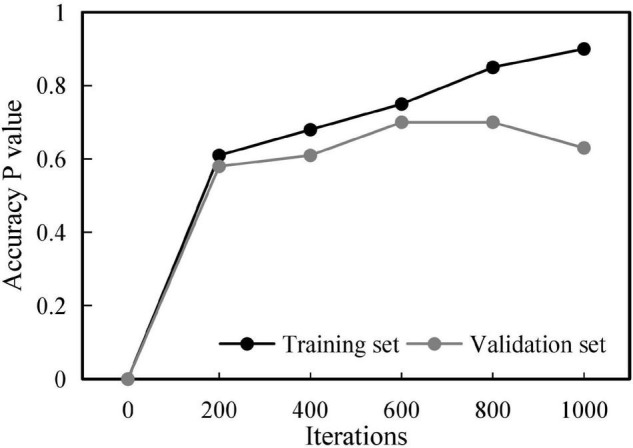
Influence of iterations on the model.

Considering that the number of artificially labeled training samples is small, if the number of iterations is too small, the learning effect will be not ideal; if the number of iterations is too large, the model overfitting problem will occur. To verify the effect of the number of iterations on the classification effect, this section divides the original training set into training set (90%) and validation set (10%) for cross-validation. The results are shown in Figure 3.

In the training set, increasing the number of iterations can effectively improve the classification accuracy, up to more than 95%. However, in the validation set, increasing the number of iterations, the classification accuracy first goes through a rapid rise stage and then begins to decline gradually. The accuracy peak of the validation set occurs at the 550th iteration. Therefore, the model at the 550th iteration is adopted as the final test model.

**FIGURE 4:**
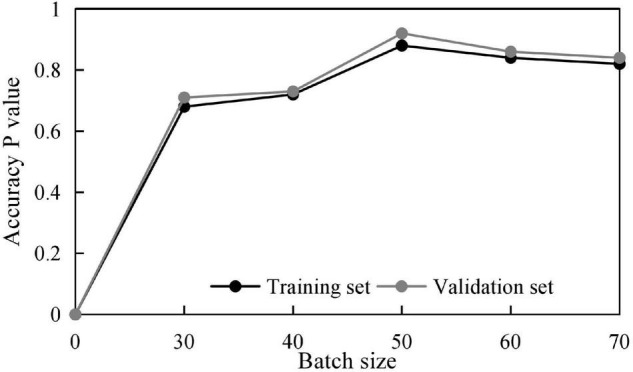
Training accuracy at different batch sizes.

**FIGURE 5:**
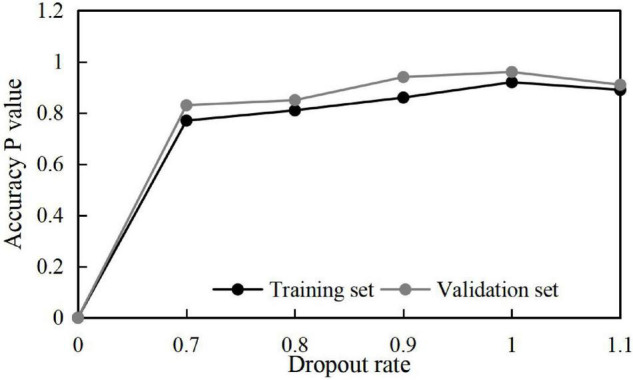
Training accuracy at different Dropout rates.

Meanwhile, to obtain some optimal parameters, the influences of different batch sizes and dropout rates on the training results are discussed, and the results shown in Figures 4, 5 are obtained.

Therefore, combining the above optimization training results, some parameters are set as shown in Table 2.

### Experimental Results and Analysis

For the test of the application effect of introducing the LIWC dictionary, this section compares the LIWC–CNN model with the traditional CNN model and compares and analyzes the LIWC–CNN model with the model established by Yates et al., so as to test the validity of the LIWC–CNN model.

1. Traditional CNN model. The first is to preprocess the text and establish a Gaussian distribution word vector matrix with a mean μ = 0 and SD δ = 1.0. The second is to perform the operation process of each level. Finally, the classification prediction results are obtained.
2. FastTextf model. The original word vector text matrix is averaged to obtain the hidden layer, and the Softmax layer in the model outputs the final classification prediction results (Shumaly et al., 2021).
3. CNN model based on Word2vec. The trained Word2Vec word vector model is initialized. Then, under the cooperative operation of the convolution layer, pooling layer, fully connected layer, and Softmax layer, the classification prediction results are obtained (Liu et al., 2020).

Using the same source data, the test results of different models are listed in Table 3. By comparison, it is found that the effectiveness of the LIWC-CNN model is the best, and its Noon-greenF1 value is equal to 0.39, which is significantly 0.33 higher than that of the traditional CNN model and 0.35 higher than the CNN model based on Word2Vec. When its UrgentF1 value is equal to 0.54, it is significantly 0.46 higher than traditional CNN.

**TABLE 3:** Comparison of experimental results of various methods.

Methods	Non-green F1	Flagged		Urgent		All	
				
		Fl	Acc	Fl	Acc	Fl	Acc
FastText	0.21	0.82	0.89	0.00	0.00	0.37	0.66
CNN	0.34	0.79	0.73	0.50	0.36	0.47	0.67
CNN + Word2Vec	0.38	0.83	0.81	0.55	0.69	0.49	0.67
LIWC-CNN	0.40	0.77	0.85	0.59	0.76	0.51	0.69

Combined with Table 3 above, the analysis is as follows:

1. The classification effect of FastText model for posts with mental health problems is not ideal. The FastText model has the highest recognition accuracy of 0.89 for flugged samples, which is the highest score among all models, but it has only 0.21 recognition accuracy for non-green samples and is not outstanding on other indicators. In addition, flagged samples exist relative to green samples, but the two kinds of samples are very uneven. The FastText model mostly attributes posts to the relatively rich green type of training samples, which indirectly affects the FastText model for the recognition effect of urgent class samples.
2. The application performance of the traditional CNN model can be significantly improved by using pre-trained Word2Vec in the traditional CNN model. All the evaluated indexes are better than those of the traditional CNN model, confirming that Word2Vec word vector features can describe the semantics of short texts more accurately and profoundly. Then the users’ psychological health status is explored. Generally speaking, Word2Vec word vector is obtained by training a language model with a full data set, which integrates richer semantic information and can realize fast convergence.
3. LIWC-CNN can efficiently and accurately identify posts that require urgent intervention. The Non-green F1 value as well as UrgentF1 value reach the highest level. At this time, the model has the best performance, but it is not as good as LIWC-CNN model.

To sum up, after introducing the LIWC dictionary into the CNN model, the new model can efficiently and accurately identify the posts with different mental health characteristics, especially the recognition effect for the post needing continued intervention is good. It can be seen that the psychological knowledge has guiding significance in the process of deep learning feature extraction, and also shapes the main advantages of the LIWC–CNN model. In essence, the LIWC–CNN model is weighted to represent the word vector in the input layer, and then the convolution kernel is called to extract the deeper features. Under this mechanism, after updating model parameters in each round of back propagation, the weight of the input word vector is adjusted, so as to achieve the maximum application effect of LIWC, which makes the model better study the psychological characteristics reflected by the text.

Overall, the best evaluation result of each model is that the non-greenF1 value is less than 0.4, which is obviously lower than the evaluation model based on the multi-feature fusion method. The main reason is that the number of samples obtained in this paper is insufficient. By using deep learning methods to construct mental health evaluation models, the psychological characteristics contained in the text can be extracted by optimizing the parameters of network structure so as to effectively avoid the tedious process of traditional methods. However, the size of the data set puts forward a higher requirement. If the number of data sets is insufficient, it will affect the evaluation effect. Combined with the reality of the scene, the number of posts in urgent need of intervention is not many, which has become a major problem constraining the promotion and application of the LIWC–CNN model.

## Conclusion

The idea of modeling in this article is to determine the weight of each word class in the LIWC lexicon in identifying different mental health problem samples based on its distribution differences in different categories of samples, which is used to guide the CNN to accurately extract the valuable semantic information in the posts and facilitate a more efficient and accurate assessment of the user’s mental health status. The experimental results confirm that the proposed method can effectively identify posts with mental health problems, which is attributed mainly to the fact that the CNN takes into account both the semantic information of the text and the semantic information of the words in the posts when modeling the content of the posts, which also creates the comparative advantage of the model in this manuscript.

## Data Availability Statement

The original contributions presented in this study are included in the article/supplementary material, further inquiries can be directed to the corresponding author/s.

## Author Contributions

All authors participated in the preparation and presentation of the manuscript and approved the submitted version.

## Conflict of Interest

The authors declare that the research was conducted in the absence of any commercial or financial relationships that could be construed as a potential conflict of interest.

## Publisher’s Note

All claims expressed in this article are solely those of the authors and do not necessarily represent those of their affiliated organizations, or those of the publisher, the editors and the reviewers. Any product that may be evaluated in this article, or claim that may be made by its manufacturer, is not guaranteed or endorsed by the publisher.
